# An outcome-wide analysis of bidirectional associations between changes in meaningfulness of life and health, emotional, behavioural, and social factors

**DOI:** 10.1038/s41598-020-63600-9

**Published:** 2020-04-15

**Authors:** Andrew Steptoe, Daisy Fancourt

**Affiliations:** Department of Behavioural Science and Health, UCL, 1-19 Torrington Place, London, WC1E 6BT UK

**Keywords:** Predictive markers, Epidemiology, Preclinical research, Risk factors

## Abstract

The sense that one is living a meaningful life is associated with positive health outcomes, but less is known about the role of changes in sense of meaning. This outcome-wide analysis investigated bidirectional associations between changes in ratings of doing worthwhile things in life and 32 factors in 6 domains of human function in 5,694 men and women (*M* = 66.65 years) from the English Longitudinal Study of Ageing. Participants rated the extent they felt that the things they did in life were worthwhile in 2012 and 2014. Analyses were adjusted for age, gender, education and social class, and were weighted for non-response. We found that health (e.g. few chronic diseases, no chronic pain), emotional wellbeing (e.g. few depressive symptoms, good sleep), greater physical activity, social factors (e.g. close relationships, friends, organizational membership, volunteering, cultural engagement), and economic factors (wealth, income), at baseline were associated with 2 year increases in worthwhile ratings. Conversely, increases in worthwhile ratings over 2 years were related to more favourable health, emotional, behavioural, and social changes between 2012 and 2016 independently of baseline levels. These bidirectional relationships highlight the importance of maintaining worthwhile activities at older ages.

## Introduction

A sense of meaning and purpose in life are core components of eudaimonic wellbeing and contribute to healthier aging^1^. Several longitudinal cohort studies have demonstrated that greater purpose in life is associated with longevity^2–4^, reduced risk of disability and functional impairment^5,6^, fewer sleep problems^7^ and more consistent use of preventive health services^8^. The sense that life has purpose is also associated with social integration, economic success and more resilient personal relationships at older ages^9^. Enhancement of purpose in life is an important component of many psychosocial interventions in people with serious physical illness^10^.

Meaning in life is a complex construct, and three aspects have been distinguished: coherence (the feeling that life makes sense), purpose (having goals and a direction in life), and significance (the sense that one’s life has inherent value and is worth living)^11,12^. Purpose has been measured using Ryff’s Psychological Well-Being Scales in many studies^13^, but simpler ratings are feasible in population studies. Research in this field lends itself to an outcome-wide approach in which the impact of a single exposure on multiple outcomes is assessed, as opposed to more traditional methods that investigate multiple determinants of a single outcome^14,15^. The outcome-wide strategy reduces the problems of mediation and collider stratification bias^16^, since it is less concerned with the conditioning of exposure estimates on outcomes depending on downstream exposures to potential mediators. A recent analysis of the English Longitudinal Study of Ageing (ELSA) showed that stronger beliefs that the things we do in life are worthwhile were associated with favourable health, behavioural, social, and economic outcomes over 4 years, independently of baseline measures and sociodemographic covariates^17^. Specifically, higher worthwhile ratings predicted better self-rated health, less pain, chronic disease and depressive symptoms, faster walking speed, more physical activity and fruit and vegetable consumption, better sleep, reduced rates of divorce and living alone, more close relationships, more contact with friends, involvement in organizations, and participation in cultural activities, more volunteering, less loneliness, and greater wealth.

The feeling that one is leading a meaningful life is not static but likely to vary over time with age and changes in circumstances^18–20^. It seems plausible that health problems would affect meaning and purpose in life, leading to changes in life goals, the sense that life is orderly and coherent, and curtailment in expectations about future direction in life. However, relatively few studies have examined the impact of health and social circumstances on measures of purpose in life in longitudinal analyses. Hill *et al*.^21^ observed wide variability in changes in purpose in life over a 3-year period in an analysis of the VA Normative Aging Study. Interestingly, age, health problems and personality did not predict changes. Another smaller study of changes over 5 years showed little change over time, and no association between changes in purpose and age or education^18^. By contrast, an analysis of the large Health and Retirement Study found that sense of purpose tended to decline on average over 8 years, with greater changes among less educated, older individuals who reported health problems^20^.

Our previous study evaluated associations between baseline ratings of the extent to which people regard the things they do in life are worthwhile with multiple outcomes both cross-sectionally and longitudinally over 4 years^17^. Here we took advantage of the fact that worthwhile ratings were repeated after a 2 year interval, providing the opportunity to build on existing findings by studying changes in doing meaningful activities. We used these data to investigate bidirectional associations between changes in worthwhile ratings over time and health, behavioural, social, and economic outcomes in detail. These analyses were not carried out in our previous study^17^, and we were able to study new relationships relevant to two issues:

- What factors are associated with increases and decreases in ratings that the things we do in life are worthwhile? By analysing the extent to which different factors are related to changes in worthwhile ratings independently of baseline levels and sociodemographic covariates, it may be possible to identify predictors of future meaning in life. We assessed 32 factors in 6 domains: physical health, biomarkers and physical capability, emotional wellbeing, health behaviour, social function and economic activity.
- Do changes in ratings of doing worthwhile things in life predict later outcomes? We assessed the combined associations of baseline worthwhile ratings and changes in ratings over two years with outcomes measured 4 years after baseline. If worthwhile ratings are causally implicated, then changes over the 2 year period should contribute to 4 year outcomes over and above the impact of baseline ratings. The number of outcomes was reduced to 27, but again covered the same six domains.

## Materials and Methods

### Data source

The data analysed in this report were derived from waves 6 (2012), 7 (2014), and 8 (2016) of ELSA, a nationally representative sample of men and women aged 50 and older living in the community in England. The study is described in detail elsewhere^22^, and full information is provided at http://www.elsa-project.ac.uk/. The sample is drawn from respondents to the Health Survey for England^23^, an annual cross-sectional survey designed to monitor the health of the general population. Data can be accessed from the UK Data Service. In wave 6 of ELSA, 7,427 respondents provided ratings of doing worthwhile things in their lives, of whom 5,773 repeated these assessments in wave 7 ratings. Information about two primary covariates (educational attainment and social class) was missing for 79, so the analytic sample was 5,694. However, there were missing values for some of the outcomes as detailed in Table 1, so the number involved in some analyses was smaller.

**Table 1:** Details of the study sample.

Variable	N	Mean ± SD/N (%)
Worthwhile rating (2012)	5,694	7.51 ± 2.14
Worthwhile rating (2014)	5,694	7.59 ± 2.13
Worthwhile rating change	5,694	0.08 ± 1.96
Age (yrs)	5,694	66.65 ± 8.68
Men/Women	5,694	2,529 (44.4%)/3,165 (55.6%)
Education	5,694	
Low		2,063 (36.2%)
Intermediate		1,650 (29.0%)
Higher		1,981 (34.8%)
Social class	5,694	
Routine and manual		2,010 (35.3%)
Intermediate		1,497 (26.3%)
Professional and managerial		2,187 (38.4%)
Health factors		
Self-rated health (good to excellent)	5,694	4,428 (77.8%)
Chronic diseases (n)	5,694	0.80 ± 0.87
Chronic pain	5,692	1,588 (27.9%)
Impaired basic ADLs	5,694	824 (14.5%)
Impaired instrumental ADLs	5,694	556 (9.8%0
Emotional wellbeing		
Depressive symptoms ≥4	5,653	588 (10.4%)
Enjoyment of life	5,636	9.95 ± 1.79
Life satisfaction	5,519	20.62 ± 6.27
Sleep quality (good, very good)	5,688	4,511 (79.3%)
Biomarkers		
Gait speed (m/s)	4,141	0.93 ± 0.26
Obesity	5,137	1,581 (30.8%)
Central adiposity	5,224	2,747 (52.6%)
Vitamin D	4,158	50.29 ± 23.23
C-reactive protein (≥3 mg/l)	4,100	1,003 (24.5%)
White blood cell count	4,122	6.39 ± 1.92
HDL-cholesterol below threshold	4,166	437 (10.5%)
Health behaviour		
Physical activity (MVPA ≥ 1/w)	5,693	3,904 (68.6%)
Sedentary behaviour	5,694	201 (3.5%)
Fruit and vegetable (n servings)	5,634	5.16 ± 2.28
Alcohol (units/wk)	5,650	2.92 ± 5.74
Current smoker	5,694	594 (10.4%)
Social factors		
Married or equivalent	5,693	3,910 (68.7%)
Living alone	5,694	1,308 (23.0%)
Close relationships (n)	5,694	7.59 ± 4.23
Friend contact ≥ 1/wk	5,694	3,759 (66.0%)
Organizations (n)	5,480	1.60 ± 1.42
Volunteer ≥ monthly	5,688	1,934 (34.0%)
Cultural activity ≥ few months	5,556	1,840 (33.1%)
Loneliness	5,694	1.37 ± 0.49
Economic factors		
Wealth £	5,393	411,472 ± 683,588
Income £	5,598	411.24 ± 482.38
Paid employment	5,694	1,736 (30.5%)

### Measures

#### Worthwhile ratings

Participants were asked: ‘Overall, to what extent do you feel the things you do in your life are worthwhile’? with responses made on a 11-point scale from 0 = *not at all* to 10 = *very*. This item is one of the set of wellbeing questions developed by the Office for National Statistics in the UK, and has been administered in several national surveys since 2011^24^. Measures were obtained at baseline (2012) and two years later (2014). The validity of this single item has not been tested from a psychometric perspective, as far as we are aware. Consequently, we analysed the association between this scale and items from the CASP-19, an quality of life measure designed for older people, that assess multiple aspects of meaning in life^25^. These items address issues of autonomy, self-realisation and control in life, and have previously been shown to predict survival among older people^26^. The worthwhile rating correlated with these items 0.59 in wave 6 (2012) and 0.56 in wave 7 (2014), both *p* < 0.0001.

#### Covariates

Four covariates that could not lie on the causal pathway were included in all analyses. Age was modelled as a continuous variable, and men were the reference category in the gender. Education was measured as the person’s highest educational qualification, divided into three categories: low (no qualifications), intermediate (qualifications at the end of state-regulated schooling), and higher (high school graduation up to university degree), The reference group in analyses was the no qualification category. Social class was defined using the National Statistics classification which allocates persons to 8 categories based on occupation, as detailed in https://www.ons.gov.uk/methodology/classificationsandstandards/otherclassifications/thenationalstatisticssocioeconomicclassificationnssecrebasedonsoc2010. The classification was collapsed into 3 categories: routine and manual occupations (reference category), intermediate occupations, and professional and managerial occupations

#### Health variables

Self-rated health is widely used as a measure of health status that predicts future health outcomes and all-cause mortality^27^. Participants rated their health as *excellent*, *very good*, *good*, *fair* and *poor*, and we analysed the proportion of giving ratings of excellent, very good, or good. The presence of chronic physical illness was analysed by assessing 6 physician-diagnosed conditions (coronary heart disease, stroke, cancer, diabetes, chronic lung disease and arthritis). The number of chronic diseases was analysed at baseline, while longitudinally, we analysed incident disease (whether or not the person had developed one or more of these conditions between 2012 and 2016). Chronic pain was assessed by asking respondents whether or not they were often troubled by pain, and if so, how intense it was (*mild, moderate*, or *severe*). The proportion who reported moderate or severe chronic pain was analysed. Longitudinally, we analysed incident chronic pain as the proportion who developed moderate or severe chronic pain between 2012 and 2016. Participants were questioned about the presence of impairments in 6 basic activities of daily living (ADLs, dressing, bathing or showering, walking across a room, eating such as cutting up food, using the toilet, and getting in and out of bed). We also assessed 7 more complicated instrumental ADLs (IADLs): difficulty preparing a hot meal, using a map, shopping for groceries, making telephone calls, taking medication, doing work around the house and garden, and difficulty managing money. The proportion of participants reporting one or more impaired ADL or IADL was analysed cross-sectionally, while longitudinal models analysed the proportion of people who were free of impaired ADLs and IADLs at baseline but developed one or more impairment between baseline and 2016.

#### Emotional wellbeing

There were four measures of emotional wellbeing. Depressive symptoms were measured using the 8-item Centre for Epidemiologic Studies Depression Scale (CES-D)^28^, a shortened scale with a Cronbach α of 0.78 in this sample. We used a score of ≥4 to indicate the presence of significant depressive symptoms, since this threshold have previously been validated against gold standard psychiatric interviews as reflecting clinically significant symptoms^29^. Enjoyment of life was assessed with 4 items from the CASP19 quality of life scale, as used in previous studies of health and disability^30^. Scores could range from 0–12 with higher ratings indicating greater affective wellbeing (Cronbach α = 0.70). Life satisfaction was measured using the Satisfaction with Life scale (Cronbach α = 0.70)^31^. Sleep quality was assessed with a 4-point rating, analysing the proportion of respondents who rated their sleep as good or very good.

#### Biomarkers and physical capability

Gait speed was used as an objective test of physical function that predicts future mortality among older people^32^. It was assessed in respondents aged ≥60 years with two 8-foot walking tests from a standing start. People with health conditions or disabilities that prevented walking were not eligible for the test. Gait speed (in m/s) was analysed as a continuously distributed variable. Height, weight and waist circumference (waist defined as the midpoint between the lower rib and the upper margin of the iliac crest) were measured by study nurses at baseline. Body mass index (BMI) was calculated and obesity was defined as a BMI ≥ 30. Central adiposity was measured as gender-specific clinically significant cut-points recommended by the US National Heart Lung and Blood Institute: 102 cm for men, and 88 cm for women. Weight but not waist circumference was re-measured in 2016, so longitudinal analyses were limited to obesity.

Four health-related biomarkers were measured at baseline (2012) during a home visit by a study nurse. Not all participants had a nurse visit, and blood sampling was not appropriate in all cases, so the sample sizes are smaller than for other measures. Plasma 25-hydroxyvitamin D (vitamin D) is important for bone and muscle health among older people^33^, and was analysed in Universal (U) units. C-reactive protein and white blood cell count were measured as indicators of inflammation. High sensitivity plasma C-reactive protein concentration was assayed and analysed as the proportion of individuals with values above or below 3 mg/L; an established threshold in population and clinical studies for significantly elevated levels. Results were the same when C-reactive protein was analysed as a continuous variable. Individuals with values ≥20 mg/L were excluded because high values may indicate the presence of an acute infection or serious acute illness. White blood cell count was analysed as a continuous variable in counts per 10^9^/L. White blood cell count is a marker associated with future cardiovascular disease, while also being involved in leptin expression and other aspects of inflammation^34^. Fourth, high density lipoprotein (HDL) cholesterol was assayed from both fasting and non-fasting samples. Low HDL-concentration is an important cardiovascular risk factor^35^, and participants were classified into the high risk group if they had HDL concentrations below sex-specific thresholds (<1.0 mmol/l for men and <1.2 mmol/l for women). These biomarkers were not assessed in the full ELSA sample in wave 8 so were not analysed longitudinally.

#### Health behaviour

Five health behaviours were analysed. Participants were questioned about the frequency with which they participated in mild, moderate, and vigorous physical activities, a measure that has been validated in a subsample against objective accelerometer measurements and is predictive of healthier aging^36^. We analysed the proportion who were either moderately or vigorously active ≥once per week. Sedentary behaviour was defined as being hardly ever or never active at mild, moderate and vigorous intensities. Fruit and vegetable intake were assessed using questions validated against biomarkers^37^. We combined fruit and vegetables to a single measure of number of portions per day. Alcohol consumption was calculated through summing the measures of spirits, glasses of wine, and pints of beer or cider drunk in the past 7 days. Smoking was assessed at interview as currently smoking cigarettes or cigars.

#### Social variables

Participants reported whether they were married/in a stable relationship, never married, divorced or separated, or widowed. The proportion of married people was analysed at baseline, and in longitudinal analysis we assessed the proportion who were married in 2012 but divorced/separated in 2016. Respondents also indicated how many people lived in their households to derive a measure of living alone. Number of close relationships was calculated by summing the number of children, other family or friends with whom respondents had a close relationship. The maximum number in each category was censored at 10, so scores could range from 0 to 30. Amount of contact with friends was assessed by asking participants if they had any friends, and if so, how much contact they had either face to face, by telephone, writing, email, or by text message. We analysed the proportion who had contact at least weekly, but similar results emerged with other cut-points for frequency of contact with friends. Respondents were also asked if they belonged to 8 different types of organization, club or society such an environmental group, residents’ association, church, or social club. The total number was analysed at both baseline and 4 years later. We measured volunteering as a prosocial behaviour and analysed the proportion who volunteered at least once per month, a frequency that has been associated with future survival^38^. Participants were asked how frequently they went to art galleries, museums, theatre, or concerts, and those who attended at least every few months were categorized as culturally active. This threshold was selected as levels below this have been shown to predict future mortality in longitudinal analyses^39^. Loneliness was measured with the three-item short form of the Revised UCLA loneliness scale^40^. Each item was scored on a 3-point scale from hardly ever or never, to some of the time, and often. Ratings were averaged to produce loneliness score ranging from 1 to 3, with higher scores indicating greater loneliness (Cronbach α = 0.83).

#### Economic variables

Wealth is a robust indicator of economic resources among older people^41^, and was measured with a detailed assessment financial, housing and physical wealth (such as land, business wealth and jewellery), excluding pension wealth. Income was computed as total weekly net family income from all sources including employment, state benefits, pensions and other assets. We analysed both wealth and income as continuous variables. The proportion of participants in paid employment (part-time or full-time) was also analysed.

### Statistical analysis

#### The determinants of changes in worthwhile ratings over two years

The change in ratings of doing worthwhile things in life were computed as 2014–2012 ratings, and regressed on the social, economic, health, biomarker, wellbeing and health behaviour measures using ordinary least squares (OLS) regression, adjusting for age, gender, education and social class. Positive regression coefficients indicate an association between the determinants and increases in worthwhile ratings. Results are presented as standardized regression coefficients (β) with standard errors and *p* values. A separate regression analysis was performed for each factor, and all analyses were weighted for nonresponse to wave 7 (2014) using inverse probability weighting (for details, see^42^). Because of multiple testing, we applied Bonferroni corrections based on the number of analyses in each domain. In the Results section, the unadjusted *p* values are presented, with indications of which effects do not reach *p* < 0.05 after correction.

#### Changes in worthwhile ratings and later outcomes

These analyses investigated associations between changes in worthwhile ratings between 2012 and 2014 and social, economic, health, biomarker, wellbeing and behavioural outcomes in 2016. All analyses took account of the baseline (2012) worthwhile ratings and the outcome variables. Binary logistic regression was used to analyse the following variables: excellent, very good or good self-rated health; incidence of one or more chronic illnesses among people who did not have this illness in 2012; incidence of impaired basic ADLs among people who did not have impaired ADLs in 2012; incidence of impaired instrumental ADLs among people who did not have impaired instrumental ADLs in 2012; significant depressive symptoms; good or very good sleep quality; obesity; moderate or vigorous physical activity (MVPA) ≥1/week; sedentary behaviour; consumption of fruit and vegetables ≥5/day; smoking; divorce by 2016 among people who were married in 2012; living alone; contact with friends weekly or more frequently; volunteering at least monthly; cultural activity every few months or more; and paid employment. The odds ratio for a unit increase in worthwhile rating (and 95% CI) are presented adjusted for covariates, with zero as the reference group. Continuously distributed variables were analysed using OLS adjusted for standard covariates plus the baseline level of the outcome variable and are presented as standardized regression coefficients (β) with standard errors and *p* values. All analyses were weighted for non-response to wave 8 (2016) using inverse probability weighting, and Bonferroni correction was applied within each domain.

In addition, we analysed the association between worthwhile ratings in 2014 and outcomes in 2016, controlling statistically for outcome values in 2014. Data were analysed using SPSS v25 and Stata SE15.

#### Sensitivity analyses

Three sensitivity analyses were conducted to explore alternative explanations of results. First, we reasoned that if people with few economic resources felt that the things they did in life became less worthwhile over time, then low affluence could play a role in any associations between changes in worthwhile ratings and social, health, emotional and behavioural factors. We therefore repeated both the analyses of the determinants of changes in worthwhile ratings, and the associations between changes in worthwhile ratings and later outcomes after including baseline wealth as an additional covariate. Second, we tested if differences in emotional distress underpinned relationships between changes in worthwhile ratings and other outcomes in another set of sensitivity analyses that included depressive symptoms as a covariate. Third, we questioned whether ceiling effects in worthwhile ratings affected these associations, given the limited scope for people rating their activities as very meaningful at baseline to show positive changes over time. These sensitivity analyses were therefore restricted to individuals with worthwhile ratings above average (i.e. ≥8) at baseline.

## Results

There were 2,529 men and 3,165 women in these analyses, ranging in age from 52 to over 90 years (mean 66.65 y) in 2012 (Table 1). Participants had relatively limited education on average with only one third attending college, and 35% had manual occupational backgrounds. Ratings of doing worthwhile things in life averaged 7.51 in 2012 and 7.59 in 2014, a small but significant rise (*p* = 0.004). Changes in worthwhile ratings were normally distributed. The distributions or average levels of health, biomarker, emotional, behavioural, social, and economic measures are shown in Table 1.

### Determinants of changes in worthwhile ratings over two years

Table 2 summarizes the regressions relating these factors with changes in worthwhile ratings. The full results of these regression analyses are summarized in Tables S1–S4 in the Supplemental material. All the measures of health were related to changes in worthwhile ratings, with larger increases among participants reporting better self-rated health, less chronic pain, and fewer basic and instrumental impaired ADLs. The association with number of chronic diseases was not significant after Bonferroni correction. The four measures of emotional wellbeing at baseline were all related to changes in worthwhile ratings over the next two years. So fewer depressive symptoms, greater enjoyment of life and life satisfaction, and better sleep quality were associated with positive changes. By contrast, few of the biomarkers measured in 2012 were associated with changes in worthwhile ratings over the next two years. However, participants with a faster gait speed at baseline showed larger increases in worthwhile ratings, as did those with better HDL-cholesterol profiles.

**Table 2:** Health, emotional wellbeing biomarkers, health behaviours, social, and economic factors in 2012 and changes in worthwhile ratings (2012–2014).

Domain	Factor (2012)	Adjusted β (SE)	*p*	Interpretation
Health	Self-rated health	0.098 (0.014)	<0.001	Better health ~ increase in ratings
	Chronic diseases (n)	−0.032 (0.015)	0.030^†^	More disease ~ decrease in ratings
	Chronic pain	−0.047 (0.014)	0.001	Chronic pain ~ decrease in ratings
	Impaired basic ADLs	−0.050 (0.014)	<0.001	Impaired ADLs ~ decrease in ratings
	Impaired IADLs	−0.043 (0.014)	0.002	Impaired IADLs~ decrease in ratings
Emotional wellbeing	Depressive symptoms	−0.104 (0.014)	<0.001	Depressive symptoms ~ decrease in ratings
	Enjoyment of life	0.250 (0.016)	<0.001	More enjoyment ~ increase in ratings
	Life satisfaction	0.232 (0.017)	<0.001	Greater satisfaction ~ increase in ratings
	Sleep quality	0.060 (0.014)	<0.001	Better sleep ~ increase in ratings
Biomarkers/physical capability	Gait speed	0.099 (0.018)	<0.001	Faster walking ~ increase in ratings
	Obesity	−0.011 (0.015)	0.46	
	Central adiposity	−0.022 (0.015)	0.15	
	Vitamin D	0.011 (0.016)	0.49	
	C-reactive protein ≥ 3 mg	0.009 (0.016)	0.57	
	White blood cell count	−0.017 (0.016)	0.30	
	HDL – cholesterol (%)	−0.044 (0.016)	0.006	Better HDL ~ increase in ratings
Health behaviour	MVPA ≥ 1/wk	0.041 (0.014)	0.004	More activity ~ increase in ratings
	Sedentary behaviour	−0.029 (0.014)	0.029^†^	More sedentary ~ decrease in ratings
	Fruit and vegetables	0.043 (0.012)	<0.001	More fruit/vegetables ~ increase in ratings
	Alcohol (units/wk)	−0.015 (0.014)	0.28	
	Smoking	−0.003 (0.014)	0.81	
Social factors	Marital status	0.061 (0.014)	<0.001	Marriage ~ increase in ratings
	Living alone	−0.044 (0.014)	0.002	Living alone ~ decrease in ratings
	Close relationships (n)	0.063 (0.014)	<0.001	More relationships ~ increase in ratings
	Friend contact ≥ 1/wk	0.028 (0.014)	0.047^†^	Frequent contact ~ increase in ratings
	Organizations (n)	0.078 (0.015)	<0.001	More organizations ~ increase in ratings
	Volunteer ≥ monthly	0.046 (0.014)	0.001	Volunteering ~ increase in ratings
	Cultural activity ≥ few months	0.039 (0.015)	0.007	More activity ~ increase in ratings
	Loneliness	−0.156 (0.015)	<0.001	Loneliness ~ decrease in ratings
Economic factors	Wealth	0.029 (0.012)	0.019^†^	Greater wealth ~ increase in ratings
	Income	0.038 (0.012)	0.002	Higher income ~ increase in ratings
	Paid employment	0.032 (0.016)	0.051	

^†^Not significant following Bonferroni correction.

Analyses adjusted for age, gender, education and social class, and weighted for non-response.

As regards health behaviours, individuals who were physically active and who ate more fruit and vegetables experienced greater increases in the sense that the things they did in life were worthwhile. There were no significant associations with alcohol intake, smoking status, or sedentary behaviour after Bonferroni correction.

All of the baseline social and relationship factors predicted changes in worthwhile ratings over two years independently of baseline worthwhile levels and covariates. Thus greater increases in ratings that life is worthwhile were associated with being in a marital relationship, not living alone, having a greater number of close relationships, membership of more organizations, greater cultural activity and lower loneliness. The association with contact with friends was not significant after Bonferroni correction. In the economic domain, greater income in 2012 was related to increases in worthwhile ratings between 2012 and 2014, but the association with employment status was not significant, and the link with wealth did not survive Bonferroni correction.

In sensitivity analysis, we tested whether the inclusion of wealth in the models attenuated associations between the various factors tested and changes in worthwhile ratings. Of the 20 associations in Table 2 with a significant Bonferroni corrected *p* value, all remained significant when wealth was added to the regression models, so the net effect of including wealth as a covariate was small.

The second set of sensitivity analyses assessed whether the association between depressive symptoms and changes in worthwhile ratings drove the other associations. One of the significant associations seen in Table 2 was no longer reliable, that for impaired IADLs (*β* = −0.027, s.e. 0.014, *p* = 0.060), but all others were unchanged.

A further sensitivity analysis tested whether associations were maintained when analyses were restricted to individuals with high worthwhile ratings at baseline. The sample size was reduced to a maximum of 4,174. Nevertheless, the results summarized in table S5 indicate that 15 of the associations were robust.

### Changes in worthwhile ratings and later outcomes

The analyses of the contribution of changes in worthwhile ratings between 2012 and 2014 to the prediction of outcomes 2 years later are summarized in Table 3. The full regression models are detailed in Tables S6–S8. Over and above the association between baseline worthwhile ratings, baseline levels of the outcomes, and covariates, changes in worthwhile ratings were independently associated with several health, emotional, behavioural and social outcomes. Thus changes in worthwhile ratings were related longitudinally with self-rated health, incident chronic pain, and ADLs in 2016, such that participants who increased worthwhile ratings reported better self-rated health, less pain and fewer impaired ADLs after adjustment for covariates. These associations did not substitute for the relationship between baseline worthwhile ratings and changes in the outcomes (as shown in the full regression models), but added to the strength of predictions.

**Table 3:** Changes in worthwhile ratings (2012–2014) and outcomes in 2016.

Domain	Factor in 2016	Odds ratio (95% CI) for change in worthwhile rating	Adjusted β (SE) for change in worthwhile rating	P
Health	Self-rated health	1.11 (1.06–1.16)		<0.001
	Incident chronic disease	0.95 (0.88–1.02)		0.15
	Incident chronic pain	0.88 (0.83–0.93)		<0.001
	Impaired basic ADLs	0.89 (0.84–0.95)		<0.001
	Impaired IADLs	0.86 (0.82–0.90)		<0.001
Emotional wellbeing	Depressive symptoms	0.79 (0.75–0.83)		<0.001
	Enjoyment of life		0.177 (0.013)	<0.001
	Life satisfaction		0.172 (0.013)	<0.001
	Sleep quality	1.13 (1.08–1.18)		<0.001
Biomarkers	Gait speed		0.045 (0.017)	<0.001
	Obesity	0.97 (0.91–1.04)		0.35
Health behaviour	MVPA ≥ 1/wk	1.11 (1.07–1.16)		<0.001
	Sedentary behaviour	0.88 (0.82–0.94)		<0.001
	Fruit/vegetables		0.078 (0.015)	<0.001
	Alcohol (units/wk)		0.004 (0.013)	0.75
	Smoking	0.97 (0.89–1.05)		0.43
Social factors	Divorce^1^	0.77 (0.66–0.89)		<0.001
	Living alone	0.83 (0.78–0.89)		<0.001
	Close relationships (n)		0.058 (0.014)	<0.001
	Friend contact ≥ 1/wk	1.03 (0.99–1.07)		0.20
	Organizations (n)		0.045 (0.013)	0.001
	Volunteer ≥ monthly	1.06 (1.01–1.11)		0.028^†^
	Culture ≥ few months	1.08 (1.03–1.14)		0.002
	Loneliness		−0.148 (0.013)	<0.001
Economic factors	Wealth		0.037 (0.012)	0.002
	Income		0.020 (0.015)	0.17
	Paid employment	1.10 (1.04–1.17)		0.002

^1^Among those married in 2012.

^†^Not significant following Bonferroni correction.

Analyses adjusted for age, gender, education and social class, and baseline levels of the outcome variable, weighted for non-response.

There were also very consistent associations with emotional wellbeing. Participants who increased worthwhile ratings between 2012 and 2014 were less likely to report fewer depressive symptoms, experienced greater enjoyment of life and life satisfaction, and had better sleep in 2016 independently of baseline levels. Only two biomarkers were available in 2016, since bloods were not drawn from the full cohort. Changes in worthwhile ratings were related to gait speed in 2016, with less reduction in gait speed among participants whose ratings increased.

The analyses of health behaviours indicated that increases in worthwhile ratings were significantly associated with an increased likelihood of engaging in moderate of vigorous physical activity and eating at least 5 portions of fruit and vegetables per day, and with lower risk of sedentary behaviour. By contrast, there were no associations with alcohol consumption or smoking in 2016, after baseline levels of these behaviours were taken into account.

In the social domain, individuals who reported an increase in the meaningfulness of their everyday lives were less likely to divorce two years later (if they were married at baseline), less likely to live alone, enjoyed more close relationships, belonged to more organizations, were more likely to engage in cultural activities, and were less lonely in 2016 than people whose worthwhile ratings declined over time.

There was a significant association between changes in worthwhile ratings and wealth but not income two years later, and changes were also related to being in paid employment in 2016.

An additional analysis tested the relationship between worthwhile ratings in 2014 and outcomes in 2016, controlling statistically for levels of the outcome variables in 2014 (Table 4). The pattern of results was similar to that shown in Table 3, with 2014 worthwhile ratings predicting 2016 self-rated health, incident chronic pain, impaired ADLs and IADLs, depressive symptoms, enjoyment of life, life satisfaction, sleep quality, gait speed, physical activity and sedentary behaviour, fruit and vegetable consumption, risk of divorce, living alone, number of close relationships, membership of organizations, cultural engagement and loneliness. In addition, 2014 worthwhile ratings were associated with wealth and income in 2016 independently of wealth and income in 2014, a result that was not evident in the analysis of changes in worthwhile ratings between 2012 and 2014.

**Table 4:** Worthwhile ratings in 2014 and changes in outcomes from 2014 to 2016.

Domain	Factor in 2016	Odds ratio (95% CI) for change in worthwhile rating	Adjusted β (SE) for change in worthwhile rating	P
Health	Self-rated health	1.13 (1.09–1.18)		<0.001
	Incident chronic disease	0.97 (0.92–1.02)		0.22
	Incident chronic pain	0.89 (0.83–0.96)		0.003
	Impaired basic ADLs	0.87 (0.81–0.94)		0.001
	Impaired IADLs	0.83 (0.79–0.88)		<0.001
Emotional wellbeing	Depressive symptoms	0.77 (0.73–0.80)		<0.001
	Enjoyment of life		0.180 (0.014)	<0.001
	Life satisfaction		0.124 (0.014)	<0.001
	Sleep quality	1.17 (1.13–1.21)		<0.001
Biomarkers	Gait speed		0.070 (0.015)	<0.001
	Obesity	0.97 (0.90–1.03		0.30
Health behaviour	MVPA ≥ 1/wk	1.10 (1.06–1.14)		<0.001
	Sedentary behaviour	0.87 (0.82–0.93)		<0.001
	Fruit/vegetables		0.074 (0.014)	<0.001
	Alcohol (units/wk)		−0.021 (0.013)	0.096
	Smoking	0.95 (0.86–1.05)		0.30
Social factors	Divorce^1^	0.86 (0.78–0.95)		0.002
	Living alone	0.76 (0.78–0.89)		<0.001
	Close relationships (n)		0.076 (0.015)	<0.001
	Friend contact ≥ 1/wk	1.05 (1.01–1.09)		0.010^†^
	Organizations (n)		0.033 (0.012)	0.008
	Volunteer ≥ monthly	1.04 (0.99–1.08)		0.12
	Culture ≥ few months	1.07 (1.02–1.12)		0.005
	Loneliness		−0.077 (0.014)	<0.001
Economic factors	Wealth		0.022 (0.011)	0.046^†^
	Income		0.037 (0.012)	0.003
	Paid employment	1.00 (0.94–1.07)		0.99

^1^Among those married in 2012.

^†^Not significant following Bonferroni correction.

Analyses adjusted for age, gender, education and social class, and baseline levels of the outcome variable, weighted for non-response.

The first two sensitivity analyses explored whether economic resources (wealth) or emotional distress (depressive symptoms) accounted for the observed associations between changes in worthwhile ratings between 2012 and 2014 and outcomes in 2016. There were no changes in the significance of the relationships detailed in Table 3 when these variables were added to the regression models (results not shown). The sensitivity analyses of people with high baseline worthwhile ratings are detailed in table S9. They show that four of the associations between changes in worthwhile ratings and social outcomes two years later (divorce, number of close relationships, volunteering and cultural engagement) were no longer significant, indicating that ceiling effects in baseline ratings may have been operating. Similarly, changes in worthwhile ratings were no longer related to future paid employment. However, all the associations with health, emotional and biomarker outcomes were unchanged from those in the complete sample, as were relationships with physical activity and diet.

## Discussion

These analyses explored the bidirectional associations between changes in feelings that one is doing worthwhile things in life and factors related to health, emotional wellbeing, behavioural, social and economic processes. Using a large population sample, we found that increases in worthwhile ratings over a 2 year period were predicted by good health and lack of disability, greater emotional wellbeing, physical activity, and a range of social factors at baseline, including marital status, living arrangements, social activity and cultural activity, and by economic factors (wealth and income). There were few associations with biomarkers. Conversely, changes in worthwhile ratings between 2012 and 2014 predicted the majority of health, emotional, behavioural and social outcomes in 2016, independently of baseline levels of these factors and baseline worthwhile ratings. In addition, worthwhile ratings in 2014 were associated with changes in 18 of the 27 outcomes analysed, replicating findings for 2012 and 2014^17^. All the analyses were controlled for age, gender, education and social class, and were weighted for non-response. Sensitivity analyses indicated that neither wealth nor emotional distress drove the broader associations.

An important consideration in this study was to test the value of a single rating of doing worthwhile things in life, rather than more complex measures of eudaimonic or hedonic wellbeing. The advantage of a single rating is that it can be included in large-scale surveys where space for different items is at a premium. This was one motive behind the development of this rating by the UK Office for National Statistics^43^. The rating has been included in a wide range of national surveys in the UK, including the Annual Population Survey, the Wealth and Assets Survey, the Crime Survey for England and Wales, the Time Use Survey, the Youth Social Activity Survey, the Life Opportunities Survey, the Armed Forces Continuous Attitudes Survey, the English Housing Survey, the Community Life Survey, the National Survey for Wales, the National Survey on People and the Natural Environment, the Living Well Index, the Active Lives Survey, and others. It has consequently been completed several million citizens over the past decade, and is used to monitor the impact of public policy on wellbeing. However, probably because the rating comes from the policy area, it has not been subject to standard psychometric evaluations of reliability and validity. In this study, we compared responses on the rating to assessments on a more established multiple item inventory, and the correlation was substantial. However, the absence of reliability data is a limitation that should be rectified in the future.

Ratings of doing worthwhile things in life likely tap into the purpose component of meaning in life, because people with valued goals in life will engage in activities that are orientated towards these goals, and these activities are therefore perceived to be more worthwhile^12^. We observed a small average increase in worthwhile ratings across the two years of assessment. Findings among older community samples have varied across studies, with no change in average ratings of purpose over 3 to 5 year intervals^18,21^, contrasting with mean reductions over longer periods^19,20^.

In our previous study, we demonstrated that higher ratings of doing worthwhile things in life were associated with a wide range of social, health, and behavioural outcomes 4 years later, independent of baseline levels of these variables^17^. The present analyses add to these findings in showing that over and above baseline worthwhile ratings, increases in ratings over a 2 year period were associated with the same set of outcomes at 4 years. It is notable that the associations with baseline worthwhile ratings were maintained after change scores were introduced into the models, so both sets of factors are relevant (Tables S6–S8). Further, they provide further information about the directionality of the relationships.

Our results indicate that good health is an important precursor to the capacity to live a meaningful life at older ages, and that in addition an increase in the sense that one is doing worthwhile things in life is associated with subsequent health and reduced risk of disability and chronic pain. This finding contrasts with an earlier analysis of a cohort of older men that showed no association between a binary measure of whether or not respondents had health problems and changes in purpose over 3 years^21^. Differences in the detail with which health was assessed may be responsible for this discrepancy. It is striking that the majority of biomarkers did not predict changes in worthwhile ratings, which suggests that the cross-sectional associations previously observed may be a result of the impact of leading a meaningful life on biology rather than *vice versa*^17^. Nevertheless, some research indicates that interventions enhancing eudaimonic wellbeing promote biological processes such as increased expression of antiviral and antibody-related gene expression^44^.

The sense that one is doing worthwhile things in life is at the heart of eudaimonic wellbeing. In these analyses, we demonstrated strong bidirectional associations between changes in worthwhile ratings and affective wellbeing (enjoyment of life) and evaluative wellbeing (life satisfaction), but these concepts show substantial overlap^45^. However, the observation in sensitivity analyses that associations between changes in worthwhile ratings and other factors were independent of depressive symptoms confirms previous findings that positive wellbeing is not merely the absence of distress and negative feeling states^46^.

The analyses of health behaviours provide evidence of a bidirectional relationship between worthwhile ratings and physical activity, in line with previous work^47,48^. We also found that improvements in worthwhile ratings predicted higher fruit and vegetable consumption. This extends existing research suggesting that improved diet is related to future wellbeing^49^. However, for health-risk behaviours such as excessing alcohol and smoking, there was no relationship.

The results suggest a bidirectional relationship between feeling that activities are worthwhile and both objective social factors (such as marital status and community engagement) and subjective social factors (such as loneliness). The only social factor for which there was no evidence of a bidirectional relationship was frequency of contact with friends. This suggests that it is productive social activities that are most clearly linked with a sense of life being worthwhile, which could perhaps be due to a sense of reward for involvement in such activities^50,51^. Our results showed that being married was associated with increases in wellbeing, contradicting an analysis of 8 year trajectories of sense of purpose in life in the Health and Retirement Study which found that marriage predicted deterioration in purpose over time^20^. This discrepancy could be due to differences in focus (perceived significance of activities vs sense of purpose), or methods, as our results were observed over a shorter period of 2 years, during which ratings of living a worthwhile life increased on average.

It is perhaps unsurprising that wealth and income predicted increases in worthwhile ratings, but that the reverse effects were less consistent. Having greater economic resources may increase opportunities to engage in activities that the individual feels are worthwhile, but feeling that one’s activities are becoming more worthwhile does not in itself lead to increases in income. It is interesting that improvements in worthwhile ratings did relate to the likelihood of having paid employment 2 years later. Previous studies have shown how improvements in emotional competencies can improve employability and entrepreneurial self-efficacy^52,53^. There is also evidence that greater sense of purpose, related as it is to goal focus, predicts future economic success^54^. The results here suggest that improvements in other aspects of eudemonic wellbeing could have similar benefits.

This study benefitted from its use of a large, nationally-representative sample of older adults and repeated measures of worthwhile ratings and behaviours. However, as the study is observational, causality cannot be assumed. We took an outcome-wide approach to statistical analysis, with a single exposure (worthwhile ratings) being related to multiple outcomes^14^. This method contrasts with the ‘exposure-wide’ strategy in which the association between worthwhile ratings and an outcome (e.g. a latent variable for physical capacity) would be tested with numerous covariates (e.g. BMI, health status, health behaviours) included in the model. A problem with the latter approach is that the association estimate will only represent a direct effect of the exposure on outcomes that does not operate through other exposures in the model, and this may underestimate the importance of meaning in life, as well as leading to potential statistical difficulties. Hence we controlled for core confounders, fixed effects derived from earlier in life (education, social class), but not factors that could lie on the causal pathway of any particular exposure-outcome relationship (such as social behaviours in models involving health-related outcomes). Nevertheless, we were able, through sensitivity analyses, to identify the independence of these results from mental ill-health and to confirm that socio-economic status does not appear to drive associations. As a further limitation, our analyses focused on a single simple measure of how worthwhile a person felt their activities were. Future studies might be able to identify whether results differ depending on the factors that have led individuals to self-rate their life as worthwhile.

Overall, the analyses of the determinants of worthwhile rating changes are consistent with the notion that a meaningful life is sustained by close interpersonal relationships, by broader social engagement, by good health, and by other aspects of subjective wellbeing. Further, here we also show that if a sense of meaning can be increased, this is associated with higher levels of health, hedonic wellbeing, some health behaviours, social behaviours, and employment. The findings support the growing consensus on the importance of prioritizing policies and public health interventions that seek to improve the wellbeing of older adults.

### Data availability

The data analysed in this article are available from the UK Data Service (accession GN 33368) at https://www.ukdataservice.ac.uk.

## Supplementary information

Supplementary information.
