# Supplementary material for: An outcome-wide analysis of bidirectional associations between changes in meaningfulness of life and health, emotional, behavioural, and social factors
Source: Sci Rep. 2020 Apr 15;10:6463. doi: 10.1038/s41598-020-63600-9 (PMC7160137; doi:10.1038/s41598-020-63600-9)
Supplement: Supplementary file 1 — Supplementary information. [file 41598_2020_63600_MOESM1_ESM.docx]

**An outcome-wide analysis of bidirectional associations between changes in meaningfulness of life and health, emotional, behavioural, and social factors**

**Andrew Steptoe and Daisy Fancourt**

**Department of Behavioural Science and Health**

**University College London**

**Supplementary Information**

**Supplementary table 1**

**Regressions of changes in worthwhile ratings on economic and health factors**

| **Factor** | **Adjusted β (SE)** | ***P*** | **Factor** | **Adjusted β (SE)** | ***P*** |
| --- | --- | --- | --- | --- | --- |
| *Wealth* |  |  | *Income* |  |  |
| Age | -0.076 (0.012) | <0.001 | Age | -0.063 (0.012) | <0.001 |
| Sex | 0.003 (0.012) | 0.80 | Sex | 0.004 (0.012) | 0.72 |
| Education | 0.004 (0.014) | 0.75 | Education | 0.006 (0.013) | 0.63 |
| Social class | 0.022 (0.013) | 0.096 | Social class | 0.021 (0.013) | 0.12 |
| 2012 worthwhile | -0.460 (0.012) | <0.001 | 2012 worthwhile | -0.461 (0.012) | <0.001 |
| Wealth | 0.029 (0.012) | 0.019 | Income | 0.038 (0.012) | 0.002 |
| *Paid employment* |  |  | *Self-rated health* | |  |
| Age | -0.050 (0.016) | <0.001 | Age | -0.059 (0.014) | <0.001 |
| Sex | -0.003 (0.014) | 0.82 | Sex | -0.007 (0.014) | 0.62 |
| Education | 0.010 (0.015) | 0.52 | Education | 0.001 (0.015) | 0.97 |
| Social class | 0.029 (0.015) | 0.058 | Social class | 0.018 (0.015) | 0.23 |
| 2012 worthwhile | -0.461 (0.014) | <0.001 | 2012 worthwhile | -0.481 (0.014) | <0.001 |
| Paid employment | 0.032 (0.016) | 0.051 | Self-rated health | 0.098 (0.014) | <0.001 |
| *Chronic disease (n)* |  |  | *Chronic pain* | |  |
| Age | -0.058 (0.014) | <0.001 | Age | -0.064 (0.014) | <0.001 |
| Sex | -0.005 (0.014) | 0.72 | Sex | -0.001 (0.014) | 0.92 |
| Education | 0.000 (0.015) | 0.56 | Education | 0.007 (0.015) | 0.66 |
| Social class | 0.026 (0.015) | 0.083 | Social class | 0.025 (0.015) | 0.98 |
| 2012 worthwhile | -0.438 (0.014) | <0.001 | 2012 worthwhile | -0.466 (0.014) | <0.001 |
| Chronic disease | -0.032 (0.015) | 0.030 | Chronic pain | -0.047 (0.014) | 0.001 |
| *Impaired basic ADLs* |  |  | *Impaired IADLs* | |  |
| Age | -0.059 (0.014) | <0.001 | Age | -0.061 (0.014) | <0.001 |
| Sex | 0.005 (0.014) | 0.73 | Sex | -0.014 (0.014) | 0.78 |
| Education | 0.007 (0.015) | 0.66 | Education | 0.007 (0.015) | 0.66 |
| Social class | 0.025 (0.015) | 0.094 | Social class | 0.025 (0.015) | 0.083 |
| 2012 worthwhile | -0.467 (0.014) | <0.001 | 2012 worthwhile | -0.466 (0.014) | <0.001 |
| Impaired ADLs | -0.050 (0.014) | <0.001 | Impaired IADLs | -0.043 (0.014) | 0.002 |

**Supplementary table 2**

**Regressions of changes in worthwhile ratings on biomarkers and physical capability measures**

| **Factor** | **Adjusted β (SE)** | ***P*** | **Factor** | **Adjusted β (SE)** | ***P*** |
| --- | --- | --- | --- | --- | --- |
| *Gait speed* |  |  | *Obesity* |  |  |
| Age | -0.027 (0.017) | 0.123 | Age | -0.057 (0.015) | <0.001 |
| Sex | -0.009 (0.016) | 0.60 | Sex | -0.005 (0.015) | 0.71 |
| Education | -0.004 (0.018) | 0.84 | Education | 0.013 (0.016) | 0.43 |
| Social class | 0.000 (0.018) | 0.99 | Social class | 0.031 (0.016) | 0.052 |
| 2012 worthwhile | -0.434 (0.016) | <0.001 | 2012 worthwhile | -0.465 (0.014) | <0.001 |
| Gait speed | 0.099 (0.018) | <0.001 | Obesity | -0.011 (0.015) | 0.46 |
| *Central adiposity* |  |  | *Vitamin D* | |  |
| Age | -0.062 (0.014) | <0.001 | Age | -0.058 (0.016) | <0.001 |
| Sex | 0.002 (0.015) | 0.89 | Sex | -0.010 (0.016) | 0.54 |
| Education | 0.013 (0.016) | 0.41 | Education | 0.003 (0.018) | 0.78 |
| Social class | 0.026 (0.016) | 0.097 | Social class | 0.008 (0.017) | 0.020 |
| 2012 worthwhile | -0.465 (0.014) | <0.001 | 2012 worthwhile | -0.477 (0.016) | <0.001 |
| Central adiposity | -0.021 (0.015) | 0.1 | Vitamin D | 0.011 (0.016) | 0.49 |
| *C-reactive protein ≥ 3 mg/l* | |  | *White blood cell count* | |  |
| Age | -0.061 (0.016) | <0.001 | Age | -0.056 (0.016) | <0.001 |
| Sex | 0.011 (0.016) | 0.49 | Sex | 0.003 (0.016) | 0.84 |
| Education | -0.002 (0.018) | 0.93 | Education | 0.007 (0.018) | 0.76 |
| Social class | 0.056 (0.018) | 0.011 | Social class | 0.040 (0.017) | 0.023 |
| 2012 worthwhile | -0.476 (0.016) | <0.001 | 2012 worthwhile | -0.477 (0.016) | <0.001 |
| C-reactive protein | 0.009 (0.016) | 0.57 | White blood cell count | -0.017 (0.016) | 0.30 |
| *HDL-cholesterol* |  |  |  | |  |
| Age | -0.059 (0.016) | <0.001 |  |  |  |
| Sex | 0.007 (0.016) | 0.68 |  |  |  |
| Education | 0.001 (0.018) | 0.94 |  |  |  |
| Social class | 0.039 (0.017) | 0.024 |  |  |  |
| 2012 worthwhile | -0.478 (0.016) | <0.001 |  |  |  |
| HDL-cholesterol | -0.044 (0.016) | 0.006 |  |  |  |

**Supplementary table 3**

**Regressions of changes in worthwhile ratings on emotional factors and health behaviours**

| **Factor** | **Adjusted β (SE)** | ***P*** | **Factor** | **Adjusted β (SE)** | ***P*** |
| --- | --- | --- | --- | --- | --- |
| *Depressive symptoms* |  |  | *Enjoyment of life* |  |  |
| Age | -0.069 (0.014) | <0.001 | Age | -0.061 (0.014) | <0.001 |
| Sex | 0.003 (0.014) | 0.82 | Sex | -0.012 (0.014) | 0.37 |
| Education | 0.008 (0.015) | 0.60 | Education | -0.004 (0.015) | 0.79 |
| Social class | 0.023 (0.015) | 0.13 | Social class | 0.015 (0.015) | 0.31 |
| 2012 worthwhile | -0.490 (0.014) | <0.001 | 2012 worthwhile | -0.590 (0.016) | <0.001 |
| Depressive symptoms | -0.104 (0.014) | <0.001 | Enjoyment of life | 0.250 (0.016) | <0.001 |
| *Life satisfaction* |  |  | *Sleep quality* | |  |
| Age | -0.070 (0.014) | <0.001 | Age | -0.068 (0.014) | <0.001 |
| Sex | 0.001 (0.014) | 0.93 | Sex | 0.000 (0.014) | 1.00 |
| Education | 0.015 (0.015) | 0.33 | Education | 0.006 (0.015) | 0.67 |
| Social class | 0.011 (0.015) | 0.45 | Social class | 0.027 (0.015) | 0.69 |
| 2012 worthwhile | -0.579 (0.017) | <0.001 | 2012 worthwhile | -0.471 (0.014) | <0.001 |
| Life satisfaction | 0.232 (0.017) | <0.001 | Sleep quality | 0.060 (0.014) | <0.001 |
| *Physical activity* |  |  | *Sedentary behaviour* | |  |
| Age | -0.061 (0.014) | <0.001 | Age | -0.064 (0.014) | <0.001 |
| Sex | -0.004 (0.014) | 0.77 | Sex | -0.006 (0.014) | 0.66 |
| Education | 0.006 (0.015) | 0.69 | Education | 0.009 (0.015) | 0.56 |
| Social class | 0.026 (0.015) | 0.087 | Social class | 0.027 (0.015) | 0.069 |
| 2012 worthwhile | -0.466 (0.014) | <0.001 | 2012 worthwhile | -0.463 (0.014) | <0.001 |
| Physical activity | 0.041 (0.014) | 0.004 | Sedentary behaviour | -0.029 (0.014) | 0.034 |
| *Fruit and vegetables* | |  | *Alcohol (units/w)* | |  |
| Age | -0.065 (0.012) | <0.001 | Age | -0.067 (0.014) | <0.001 |
| Sex | -0.004 (0.012) | 0.76 | Sex | -0.012 (0.014) | 0.42 |
| Education | 0.003 (0.013) | 0.79 | Education | 0.010 (0.015) | 0.50 |
| Social class | 0.025 (0.013) | 0.058 | Social class | 0.030 (0.015) | 0.050 |
| 2012 worthwhile | -0.465 (0.012) | <0.001 | 2012 worthwhile | -0.460 (0.014) | <0.001 |
| Fruit and vegetables | 0.043 (0.012) | <0.001 | Alcohol intake | -0.015 (0.014) | 0.28 |
| *Current smoking* |  |  |  |  |  |
| Age | -0.067 (0.014) | <0.001 |  |  |  |
| Sex | -0.006 (0.014) | 0.68 |  |  |  |
| Education | 0.010 (0.015) | 0.52 |  |  |  |
| Social class | 0.029 (0.015) | 0.058 |  |  |  |
| 2012 worthwhile | -0.460 (0.014) | <0.001 |  |  |  |
| Current smoking | -0.003 (0.014) | 0.81 |  |  |  |

**Supplementary table 4**

**Regressions of changes in worthwhile ratings on social factors**

| **Factor** | **Adjusted β (SE)** | ***P*** | **Factor** | **Adjusted β (SE)** | ***P*** |
| --- | --- | --- | --- | --- | --- |
| *Marital status* |  |  | *Living alone* |  |  |
| Age | -0.059 (0.014) | <0.001 | Age | -0.057 (0.014) | <0.001 |
| Sex | 0.003 (0.014) | 0.86 | Sex | -0.001 (0.014) | 0.96 |
| Education | 0.008 (0.015) | 0.61 | Education | 0.008 (0.015) | 0.59 |
| Social class | 0.028 (0.015) | 0.063 | Social class | 0.029 (0.015) | 0.053 |
| 2012 worthwhile | -0.469 (0.014) | <0.001 | 2012 worthwhile | -0.466 (0.014) | <0.001 |
| Marital status | 0.061 (0.014) | <0.001 | Living alone | -0.044 (0.014) | 0.002 |
| *Close relationships (n)* |  |  | *Friend contact ≥ 1/wk* | |  |
| Age | -0.068 (0.014) | <0.001 | Age | -0.067 (0.014) | <0.001 |
| Sex | -0.010 (0.014) | 0.49 | Sex | -0.008 (0.014) | 0.55 |
| Education | 0.008 (0.015) | 0.59 | Education | 0.009 (0.015) | 0.54 |
| Social class | 0.029 (0.015) | 0.052 | Social class | 0.029 (0.015) | 0.052 |
| 2012 worthwhile | -0.475 (0.014) | <0.001 | 2012 worthwhile | -0.463 (0.014) | <0.001 |
| N close relationships | 0.063 (0.014) | <0.001 | Friend contact | 0.028 (0.014) | 0.047 |
| *Organisations (n)* |  |  | *Volunteer ≥ monthly* | |  |
| Age | -0.076 (0.014) | <0.001 | Age | -0.068 (0.014) | <0.001 |
| Sex | -0.011 (0.014) | 0.43 | Sex | -0.007 (0.014) | 0.63 |
| Education | 0.001 (0.016) | 0.95 | Education | 0.005 (0.015) | 0.75 |
| Social class | 0.011 (0.016) | 0.49 | Social class | 0.024 (0.015) | 0.11 |
| 2012 worthwhile | -0.468 (0.014) | <0.001 | 2012 worthwhile | -0.462 (0.014) | <0.001 |
| Organisations | 0.078 (0.015) | <0.001 | Volunteering | -0.046 (0.014) | 0.001 |
| *Loneliness* |  |  | *Cultural activity ≥ few months* | |  |
| Age | -0.069 (0.014) | <0.001 | Age | -0.071 (0.014) | <0.001 |
| Sex | 0.012 (0.014) | 0.39 | Sex | -0.011 (0.014) | 0.45 |
| Education | 0.008 (0.015) | 0.59 | Education | 0.005 (0.016) | 0.75 |
| Social class | 0.023 (0.015) | 0.13 | Social class | 0.030 (0.016) | 0.055 |
| 2012 worthwhile | -0.515 (0.014) | <0.001 | 2012 worthwhile | -0.460 (0.014) | <0.001 |
| Loneliness | -0.156 (0.015) | <0.001 | Cultural activity | 0.036 (0.015) | 0.007 |

**Supplementary table 5**

**Health, emotional wellbeing biomarkers, health behaviours, social, and economic factors in 2012 and changes in worthwhile ratings (2012- 2014)**

**Analysis limited to participants with above average worthwhile ratings at baseline (≥8)**

| **Domain** | **Factor (2012)** | **Adjusted β (SE)** | ***p*** | **Interpretation** |
| --- | --- | --- | --- | --- |
| Social | Marital status | 0.052 (0.020) | 0.010 | Marriage ~ increase in ratings |
| factors | Living alone | -0.039 (0.020) | 0.055 |  |
|  | Close relationships (n) | 0.066 (0.020) | 0.001 | More relationships ~ increase in ratings |
|  | Friend contact ≥ 1/wk | 0.038 (0.020) | 0.055 |  |
|  | Organisations (n) | 0.092 (0.021) | <0.001 | More organisations ~ increase in ratings |
|  | Volunteer ≥ monthly | 0.058 (0.020) | 0.004 | Volunteering ~ increase in ratings |
|  | Cultural activity ≥ few months | 0.073 (0.021) | <0.001 | More activity ~ increase in ratings |
|  | Loneliness | -0.074 (0.020) | <0.001 | Loneliness ~ decrease in ratings |
| Economic | Wealth | 0.029 (0.017) | 0.092 |  |
| factors | Income quintile | 0.034 (0.017) | 0.052 |  |
|  | Paid employment | 0.019 (0.023) | 0.42 |  |
| Health | Self-rated health | 0.113 (0.020) | <0.001 | Better health ~ increase in ratings |
|  | Chronic diseases (n) | -0.039 (0.020) | 0.060 |  |
|  | Chronic pain | -0.036 (0.020) | 0.072 |  |
|  | Impaired basic ADLs | -0.031 (0.020) | 0.044† | Impaired ADLs ~ decrease in ratings |
|  | Impaired IADLs | -0.058 (0.020) | 0.004 | Impaired IADLs~ decrease in ratings |
| Biomarkers/ | Gait speed | 0.116 (0.025) | <0.001 | Faster walking ~ increase in ratings |
| physical | Obesity | -0.036 (0.021) | 0.080 |  |
| capability | Central adiposity | -0.032 (0.021) | 0.13 |  |
|  | Vitamin D | 0.034 (0.023) | 0.14 |  |
|  | C-reactive protein ≥ 3mg | -0.011 (0.023) | 0.63 |  |
|  | White blood cell count | -0.031 (0.023) | 0.18 |  |
|  | HDL – cholesterol (%) | -0.089 (0.023) | <0.001 | Better HDL ~ increase in ratings |
| Emotional | Depressive symptoms | -0.172 (0.019) | <0.001 | Depressive symptoms ~ decrease in ratings |
| wellbeing | Enjoyment of life | 0.224 (0.020) | <0.001 | More enjoyment ~ increase in ratings |
|  | Life satisfaction | 0.210 (0.020) | <0.001 | Greater satisfaction ~ increase in ratings |
|  | Sleep quality | 0.085 (0.020) | <0.001 | Better sleep ~ increase in ratings |
| Health | MVPA ≥ 1/wk | 0.050 (0.020) | 0.012† | More activity ~ increase in ratings |
| behaviour | Sedentary behaviour | -0.046 (0.020) | 0.021† | More sedentary ~ decrease in ratings |
|  | Fruit and vegetables | 0.050 (0.017) | 0.003 | More fruit/vegetables ~ increase in ratings |
|  | Alcohol (units/wk) | -0.038 (0.021) | 0.067 |  |
|  | Smoking | -0.034 (0.020) | 0.085 |  |

† = not significant after Bonferroni correction

Analyses adjusted for age, gender, education and social class, and weighted for non-response

**Supplementary table 6**

**Changes in worthwhile ratings (2014-2012) and economic and health factors in 2016**

| **Factor** | **Adjusted β (SE)** | ***P*** | **Factor** | **Adjusted β (SE)** | ***P*** | |  |
| --- | --- | --- | --- | --- | --- | --- | --- |
| *Wealth* |  |  | *Income* |  |  | |  |
| Age | -0.048 (0.011) | <0.001 | Age | -0.083 (0.014) | <0.001 | |  |
| Sex | 0.007 (0.011) | 0.53 | Sex | -0.035 (0.013) | 0.008 | |  |
| Education | 0.081 (0.012) | <0.001 | Education | 0.120 (0.015) | <0.001 | |  |
| Social class | 0.062 (0.012) | <0.001 | Social class | 0.129 (0.015) | <0.001 | |  |
| Wealth 2012 | 0.672 (0.011) | <0.001 | Income 2012 | 0.356 (0.014) | <0.001 | |  |
| Worthwhile (2012) | 0.034 (0.012) | 0.006 | Worthwhile (2012) | 0.064 (0.015) | <0.001 | |  |
| Worthwhile change | 0.037 (0.012) | 0.002 | Worthwhile change | 0.020 (0.015) | 0.17 | |  |
|  | **Adjusted odds ratio (95% CI)** | ***P*** |  | **Adjusted odds ratio (95% CI)** | ***P*** | |  |
| *Paid employment* | |  | *Self-rated health* |  |  | |  |
| Age | 0.84 (0.82-0.86) | <0.001 | Age | 0.97 (0.96-0.98) | <0.001 | |  |
| Sex | 0.62 (0.51-0.75) | <0.001 | Sex | 1.05 (0.89-1.25) | 0.54 | |  |
| Education  Lower  Intermediate  Higher | 1  0.99 (0.77-1.27)  0.99 (0.77-1.29) | 0.92  0.96 | Education  Lower  Intermediate  Higher | 1  1.25 (1.02-1.54)  1.47 (1.17-1.83) | 0.030  0.001 | |  |
| Social class  Routine  Intermediate  Professional | 1  1.31 (0.87-1.47)  0.79 (0.62-1.00) | 0.36  0.054 | Social class  Routine  Intermediate  Professional | 1  1.46 (1.18-1.80)  1.60 (1.29-1.98) | <0.001  <0.001 | |  |
| Employment in 2012 | 22.82 (17.81- 29.23) | <0.001 | Self-rated health (2012) | 12.83 (10.75-15.30) | <0.001 | |  |
| Worthwhile (2012) | 1.08 (1.03-1.14) | <0.001 | Worthwhile (2012) | 1.15 (1.10-1.20) | <0.001 | |  |
| Worthwhile change | 1.10 (1.04-1.17) | 0.002 | Worthwhile change | 1.11 (1.06-1.16) | <0.001 | |  |
| *Incident chronic disease* |  |  | *Incident chronic pain* | | |  | |
| Age | 1.04 (1.03-1.06) | <0.001 | Age | 1.02 (1.01-1.03) | <0.001 | |  |
| Sex | 0.97 (0.76-1.23) | 0.78 | Sex | 1.45 (1.19-1.78) | <0.001 | |  |
| Education  Lower  Intermediate  Higher | 1  0.78 (0.58-1.04)  0.64 (0.47-0.87) | 0.085  0.005 | Education  Lower  Intermediate  Higher | 1  0.87 (0.68-1.10)  0.74 (0.56-0.97) | 0.24  0.026 | |  |
| Social class  Routine  Intermediate  Professional | 1  0.99 (0.74-1.33)  0.71 (0.52-0.96) | 0.95  0.027 | Social class  Routine  Intermediate  Professional | 1  0.85 (0.66-1.09)  0.72 (0.56-0.93) | 0.21  0.012 | |  |
| Worthwhile (2012) | 0.90 (0.85-0.96) | <0.001 | Worthwhile (2012) | 0.90 (0.85-0.95) | <0.001 | |  |
| Worthwhile change | 0.95 (0.88-1.02) | 0.15 | Worthwhile change | 0.88 (0.83-0.93) | <0.001 | |  |
| *Incident basic ADLs* |  |  | *Incident instrumental ADLs* | | |  | |
| Age | 1.07 (1.06-1.08) | <0.001 | Age | 1.07 (1.06-1.08) | <0.001 | |  |
| Sex | 0.92 (0.72-1.18) | 0.52 | Sex | 1.58 (1.30-1.93) | <0.001 | |  |
| Education  Lower  Intermediate  Higher | 1  0.91 (0.68-1.23)  0.84 (0.61-1.16) | 0.91  0.84 | Education  Lower  Intermediate  Higher | 1  0.78 (0.61-0.99)  0.88 (0.68-1.14) | 0.044  0.34 | |  |
| Social class  Routine  Intermediate  Professional | 1  0.73 (0.53-0.99)  0.74 (0.54-0.99) | 0.047  0.049 | Social class  Routine  Intermediate  Professional | 1  0.73 (0.57-0.94)  0.67 (0.52-0.86) | 0.011  0.002 | |  |
| Worthwhile (2012) | 0.84 (0.79-0.89) | <0.001 | Worthwhile (2012) | 0.79 (0.75-0.83) | <0.001 | |  |
| Worthwhile change | 0.89 (0.84-0.95) | <0.001 | Worthwhile change | 0.86 (0.82-0.90) | <0.001 | |  |
|  |  |  |  | **Adjusted β (SE)** | ***P*** | |  |
| *Obesity* |  |  | *Gait speed* |  |  | |  |
| Age | 0.97 (0.96-0.98) | <0.001 | Age | -0.182 (0.016) | <0.001 | |  |
| Sex | 1.08 (0.86-1.34) | 0.52 | Sex | -0.018 (0.016) | 0.26 | |  |
| Education  Lower  Intermediate  Higher | 1  1.27 (0.97-1.68)  1.13 (0.84-1.52) | 0.086  0.43 | Education | 0.029 (0.017) | 0.10 | |  |
| Social class  Routine  Intermediate  Professional | 1  0.73 (0.55-0.96)  0.57 (0.43-0.75) | 0.026  <0.001 | Social class | 0.033 (0.017) | 0.056 | |  |
| Obesity (2012) | 75.39 (60.13-94.51) | <0.001 | Gait speed 2012 | 0.532 (0.017) | <0.001 | |  |
| Worthwhile (2012) | 0.93 (0.88-0.99) | 0.014 | Worthwhile (2012) | 0.061 (0.018) | 0.001 | |  |
| Worthwhile change | 0.97 (0.91-1.05) | 0.35 | Worthwhile change | 0.045 (0.017) | 0.010 | |  |

**Supplementary table 7**

**Changes in worthwhile ratings (2014-2012) and wellbeing and health behaviour in 2016**

| **Factor** | **Adjusted β (SE)** | ***P*** | **Factor** | **Adjusted β (SE)** | ***P*** |
| --- | --- | --- | --- | --- | --- |
| *Enjoyment of life* |  |  | *Life satisfaction* |  |  |
| Age | -0.072 (0.012) | <0.001 | Age | -0.030 (0.012) | 0.008 |
| Sex | 0.013 (0.011) | 0.26 | Sex | 0.006 (0.012) | 0.56 |
| Education | 0.025 (0.013) | 0.052 | Education | 0.013 (0.012) | 0.29 |
| Social class | 0.055 (0.013) | <0.001 | Social class | 0.018 (0.012) | <0.001 |
| Enjoyment of life 2012 | 0.528 (0.016) | <0.001 | Life satisfaction 2012 | 0.598 (0.016) | <0.001 |
| Worthwhile (2012) | 0.235 (0.014) | <0.001 | Worthwhile (2012) | 0.202 (0.014) | 0.011 |
| Worthwhile change | 0.177 (0.013) | <0.001 | Worthwhile change | 0.172 (0.013) | 0.087 |
|  | **Adjusted odds ratio (95% CI)** | ***P*** |  | **Adjusted odds ratio (95% CI)** | ***P*** |
| *Depressive symptoms ≥4* | |  | *Sleep quality* |  |  |
| Age | 1.02 (1.01-1.03) | 0.001 | Age | 1.01 (1.00-1.02) | 0.18 |
| Sex | 1.54 (1.23-1.92) | <0.001 | Sex | 0.67 (0.57-0.79) | <0.001 |
| Education  Lower  Intermediate  Higher | 1  0.78 (0.60-1.01)  0.64 (0.48-0.87) | 0.063  0.004 | Education  Lower  Intermediate  Higher | 1  1.29 (1.04-1.55)  1.45 (1.16-1.80) | 0.021  0.001 |
| Social class  Routine  Intermediate  Professional | 1  0.97 (0.74-1.28)  0.97 (0.73-1.28) | 0.85  0.80 | Social class  Routine  Intermediate  Professional | 1  1.28 (1.04-1.58)  1.20 (1.02-1.54) | 0.020  0.035 |
| Depression 2012 | 7.59 (5.94-9.70) | <0.001 | Sleep quality 2012 | 8.70 (7.36-10.29) | <0.001 |
| Worthwhile (2012) | 0.74 (0.70-0.78) | <0.001 | Worthwhile (2012) | 1.20 (1.15-1.25) | <0.001 |
| Worthwhile change | 0.79 (0.74-0.83) | <0.001 | Worthwhile change | 1.13 (1.08-1.18) | <0.001 |
| *Moderate-vigorous physical activity ≥1/w* | |  | *Sedentary behaviour* | |  |
| Age | 0.96 (0.95-0.97) | <0.001 | Age | 1.07 (1.06-1.09) | <0.001 |
| Sex | 0.88 (0.77-1.02) | 0.084 | Sex | 0.67 (0.51-0.89) | 0.005 |
| Education  Lower  Intermediate  Higher | 1  1.17 (0.99-1.34)  1.54 (1.28-1.86) | 0.068  <0.001 | Education  Lower  Intermediate  Higher | 1  0.71 (0.50-1.01)  0.62 (0.42-0.93) | 0.058  0.020 |
| Social class  Routine  Intermediate  Professional | 1  1.41 (1.18-1.69)  1.57 (1.31-1.87) | <0.001  <0.001 | Social class  Routine  Intermediate  Professional | 1  0.65 (0.46-0.93)  0.53 (0.37-0.78) | 0.18  0.001 |
| Physical activity 2012 | 4.37 (3.79-5.05) | <0.001 | Sedentary behaviour 2012 | 8.83 (5.95-13.10) | <0.001 |
| Worthwhile (2012) | 1.12 (1.08-1.16) | <0.001 | Worthwhile (2012) | 0.82 (0.77-0.87) | <0.001 |
| Worthwhile change | 1.11 (1.07-1.16) | <0.001 | Worthwhile change | 0.88 (0.82-0.93) | <0.001 |
|  | |  | *Smoking* | |  |
|  |  |  | Age | 0.99 (0.97-1.01) | 0.20 |
|  |  |  | Sex | 1.30 (0.94-1.80) | 0.11 |
|  |  |  | Education  Lower  Intermediate  Higher | 1  0.71 (0.48-1.04)  0.54 (0.35-0.84) | 0.078  0.007 |
|  |  |  | Social class  Routine  Intermediate  Professional | 1  0.60 (0.40-0.93)  1.01 (0.68-1.50) | 0.017  0.97 |
|  |  |  | Smoking 2012 | 189.6 (133.0-270.1) | <0.001 |
|  |  |  | Worthwhile (2012) | 0.99 (0.92-1.08) | 0.86 |
|  |  |  | Worthwhile change | 0.97 (0.89-1.05) | 0.43 |
|  |  |  |  |  |  |
|  | **Adjusted β (SE)** | ***P*** |  | **Adjusted β (SE)** | ***P*** |
| *Alcohol (units/w)* |  |  | *Fruit/vegetables* |  |  |
| Age | -0.058 (0.012) | <0.001 | Age | -0.019 (0.013) | 0.15 |
| Sex | -0.132 (0.012) | <0.001 | Sex | 0.067 (0.013) | <0.001 |
| Education | -0.027 (0.013) | 0.038 | Education | 0.024 (0.015) | 0.10 |
| Social class | -0.015 (0.013) | 0.23 | Social class | 0.018 (0.014) | 0.22 |
| Alcohol 2012 | 0.599 (0.012) | <0.001 | Fruit/vegetables 2012 | 0.515 (0.013) | <0.001 |
| Worthwhile (2012) | 0.021 (0.013) | 0.12 | Worthwhile (2012) | 0.118 (0.015) | <0.001 |
| Worthwhile change | 0.004 (0.013) | 0.75 | Worthwhile change | 0.078 (0.015) | <0.001 |

**Supplementary table 8**

**Changes in worthwhile ratings (2014-2012) and social factors in 2016**

| **Factor** | **Adjusted Odds ratio (95% CI)** | ***P*** | | **Factor** | | | **Adjusted Odds ratio (95% CI)** | | ***P*** | |  |
| --- | --- | --- | --- | --- | --- | --- | --- | --- | --- | --- | --- |
| *Divorce among married respondents* | | |  | | *Living alone* | | |  | |  | |
| Age | 0.89 (0.85-0.93) | <0.001 | | Age | | | 1.04 (1.03-1.05) | | <0.001 | |  |
| Sex | 1.53 (0.87-2.68) | 0.14 | | Sex | | | 1.90 (1.47-2.46) | | <0.001 | |  |
| Education  Lower  Intermediate  Higher | 1  0.75 (0.37-1.53)  0.79 (0.38-1.64) | 0.43  0.53 | | Education  Lower  Intermediate  Higher | | | 1  0.89 (0.66-1.22)  1.01 (0.72-1.41) | | 0.47  0.96 | |  |
| Social class  Routine  Intermediate  Professional | 1  0.85 (0.37-1.92)  1.68 (0.83-3.34) | 0.69  0.14 | | Social class  Routine  Intermediate  Professional | | | 1  0.87 (0.64-1.20)  0.74 (0.54-1.02) | | 0.40  0.068 | |  |
| Worthwhile (2012) | 0.79 (0.68-0.91) | 0.001 | | Living alone (2012) | | | 168.0 (126.3-223.0) | | <0.001 | |  |
| Worthwhile change | 0.77 (0.63-0.90) | <0.001 | | Worthwhile (2012) | | | 0.87 (0.82-0.93) | | <0.001 | |  |
|  |  |  | | Worthwhile change | | | 0.83 (0.79-0.89) | | <0.001 | |  |
| *Contact with friends* |  |  | | *Volunteering ≥ monthly* | | | | |  | |  |
| Age | 1.00 (0.99-1.01) | 0.35 | | Age | | | 1.00 (0.99-1.01) | | 0.76 | |  |
| Sex | 1.31 (1.14-1.51) | <0.001 | | Sex | | | 1.31 (1.14-1.52) | | 0.001 | |  |
| Education  Lower  Intermediate  Higher | 1  0.91 (0.76-1.08)  1.04 (0.86-1.26) | 0.68  0.28 | | Education  Lower  Intermediate  Higher | | | 1  1.05 (0.86-1.28)  1.42 (1.16-1.74) | | 0.63  0.001 | |  |
| Social class  Routine  Intermediate  Professional | 1  1.03 (0.86-1.23)  0.90 (0.73-1.08) | 0.25  0.76 | | Social class  Routine  Intermediate  Professional | | | 1  1.37 (1.12-1.69)  1.57 (1.29-1.92) | | 0.003  <0.001 | |  |
| Contact 2012 | 6.88 (5.97-7.93) | <0.001 | | Volunteering 2012 | | | 11.26 (0.9-13.15) | | <0.001 | |  |
| Worthwhile (2012) | 1.07 (1.03-1.11) | <0.001 | | Worthwhile (2012) | | | 1.05 (1.01-1.10) | | 0.018 | |  |
| Worthwhile change | 1.03 (0.99-1.07) | 0.20 | | Worthwhile change | | | 1.06 (1.01-1.11) | | 0.028 | |  |
| *Cultural activity ≥ few months* | | |  | |  | | | | |  | |
| Age | 0.98 (0.97-0.99) | <0.001 | |  | | |  | |  | |  |
| Sex | 1.03 (0.87-1.21) | 0.76 | |  | | |  | |  | |  |
| Education  Lower  Intermediate  Higher | 1  1.04 (0.84-1.29)  1.47 (1.18-1.83) | 0.73  0.001 | |  | | |  | |  | |  |
| Social class  Routine  Intermediate  Professional | 1  1.25 (1.00-1.55)  1.27 (1.03-1.57) | 0.051  0.024 | |  | | |  | |  | |  |
| Cultural activity 2012 | 13.87 (11.73-16.39) | <0.001 | |  | | |  | |  | |  |
| Worthwhile (2012) | 1.07 (1.03-1.12) | 0.002 | |  | | |  | |  | |  |
| Worthwhile change | 1.08 (1.03-1.14) | 0.002 | |  | | | | |  | |  |
|  | **Adjusted β (SE)** | ***P*** | |  | | **Adjusted β (SE)** | | | ***P*** | |  |
| *Close relationships* |  |  | | *Organisations* | |  | | |  | |  |
| Age | 0.001 (0.013) | 0.95 | | Age | | | 0.021 (0.012) | | 0.075 | |  |
| Sex | 0.027 (0.013) | 0.033 | | Sex | | | 0.020 (0.012) | | 0.077 | |  |
| Education | -0.015 (0.014) | 0.28 | | Education | | | 0.059 (0.013) | | <0.001 | |  |
| Social class | 0.095 (0.015) | <0.001 | | Social class | | | 0.024 (0.013) | | 0.060 | |  |
| Close relationships 2012 | 0.535 (0.013) | <0.001 | | Organisations 2012 | | | 0.662 (0.012) | | <0.001 | |  |
| Worthwhile (2012) | 0.095 (0.015) | <0.001 | | Worthwhile (2012) | | | 0.057 (0.013) | | <0.001 | |  |
| Worthwhile change | 0.058 (0.014) | <0.001 | | Worthwhile change | | | 0.045 (0.013) | | 0.001 | |  |
| *Loneliness* |  |  | |  | | |  | |  | |  |
| Age | 0.017 (0.012) | 0.17 | |  | | |  | |  | |  |
| Sex | 0.022 (0.012) | 0.071 | |  | | |  | |  | |  |
| Education | -0.016 (0.014) | 0.23 | |  | | |  | |  | |  |
| Social class | -0.029 (0.015) | 0.029 | |  | | |  | |  | |  |
| Loneliness 2012 | 0.560 (0.013) | <0.001 | |  | | |  | |  | |  |
| Worthwhile (2012) | -0.170 (0.015) | <0.001 | |  | | |  | |  | |  |
| Worthwhile change | -0.148 (0.013) | <0.001 | |  | | |  | |  | |  |

**Supplementary table 9**

**Changes in worthwhile ratings (2012-2014) and outcomes in 2016**

**Analysis limited to participants with above average worthwhile ratings at baseline (≥8)**

| **Domain** | **Factor in 2016** | **Odds ratio**  **(95% CI) for change in worthwhile rating** | **Adjusted β (SE) for change in worthwhile rating** | **P** |
| --- | --- | --- | --- | --- |
| Social | Divorce^1^ | 0.78 (0.62-0.99) |  | 0.044† |
| factors | Living alone | 0.81 (0.74-0.99) |  | <0.001 |
|  | Close relationships (n) |  | 0.035 (0.018) | 0.045† |
|  | Friend contact ≥ 1/wk | 1.02 (0.96-1.08) |  | 0.57 |
|  | Organisations (n) |  | 0.039 (0.016) | 0.012 |
|  | Volunteer ≥ monthly | 1.05 (0.98-1.12) |  | 0.20 |
|  | Culture ≥ few months | 1.04 (0.97-1.12) |  | 0.27 |
|  | Loneliness |  | -0.135 (0.017) | <0.001 |
| Economic | Wealth |  | 0.040 (0.015) | 0.009 |
| factors | Income |  | 0.019 (0.018) | 0.30 |
|  | Paid employment | 1.06 (0.97-1.17) |  | 0.20 |
| Health | Self-rated health | 1.11 (1.03-1.18) |  | 0.003 |
|  | Incident chronic disease | 1.02 (0.91-1.14) |  | 0.79 |
|  | Incident chronic pain | 0.89 (0.82-0.96) |  | 0.002 |
|  | Impaired basic ADLs | 0.88 (0.81-0.96) |  | 0.005 |
|  | Impaired IADLs | 0.78 (0.73-0.80) |  | <0.001 |
| Emotional | Depressive symptoms | 0.80 (0.73-0.86) |  | <0.001 |
| wellbeing | Enjoyment of life |  | 0.186 (0.018) | <0.001 |
|  | Life satisfaction |  | 0.159 (0.017) | <0.001 |
|  | Sleep quality | 1.11 (1.04-1.18) |  | 0.002 |
| Biomarkers | Gait speed |  | 0.052 (0.021) | 0.013 |
|  | Obesity | 1.03 (0.91-1.13) |  | 0.56 |
| Health | MVPA ≥ 1/wk | 1.11 (1.05-1.18) |  | <0.001 |
| behaviour | Sedentary behaviour | 0.91 (0.82-1.01) |  | 0.062 |
|  | Fruit/vegetables |  | 0.070 (0.018) | <0.001 |
|  | Alcohol (units/wk) |  | 0.004 (0.016) | 0.82 |
|  | Smoking | 0.92 (0.80-1.06) |  | 0.26 |
|  |  |  |  |  |

^1^ Among those married in 2012

† = not significant following Bonferroni correction

Analyses adjusted for age, gender, education and social class, and baseline levels of the outcome variable, weighted for non-response
